# *Phytomonas*: Trypanosomatids Adapted to Plant Environments

**DOI:** 10.1371/journal.ppat.1004484

**Published:** 2015-01-21

**Authors:** Eleanor Jaskowska, Claire Butler, Gail Preston, Steven Kelly

**Affiliations:** Department of Plant Sciences, University of Oxford, Oxford, United Kingdom; International Centre for Genetic Engineering and Biotechnology, INDIA

## Abstract

Over 100 years after trypanosomatids were first discovered in plant tissues, *Phytomonas* parasites have now been isolated across the globe from members of 24 different plant families. Most identified species have not been associated with any plant pathology and to date only two species are definitively known to cause plant disease. These diseases (wilt of palm and coffee phloem necrosis) are problematic in areas of South America where they threaten the economies of developing countries. In contrast to their mammalian infective relatives, our knowledge of the biology of *Phytomonas* parasites and how they interact with their plant hosts is limited. This review draws together a century of research into plant trypanosomatids, from the first isolations and experimental infections to the recent publication of the first *Phytomonas* genomes. The availability of genomic data for these plant parasites opens a new avenue for comparative investigations into trypanosomatid biology and provides fresh insight into how this important group of parasites have adapted to survive in a spectrum of hosts from crocodiles to coconuts.

## Introduction

The trypanosomatids are a monophyletic group of single-celled eukaryotic parasites that are spread between multicellular hosts predominantly by insects. Globally, these parasites cause a considerable burden on human health and welfare, with an estimated 20 million people infected with trypanosomatid pathogens [1], as well as several devastating diseases of livestock. Thus, trypanosomatids have attracted significant attention from disparate academic communities for their importance to global human welfare. In more recent years, trypanosomatids have also drawn interest for use as model organisms because of their streamlined and often extreme biology. In this context, the study of trypanosomatid biology has greatly contributed to our understanding of several biological phenomena, including mechanisms of immune evasion [2], glycosylphosphatidylinositol anchors [3], RNA editing [4], polycistronic transcription [5], trans-splicing [6], chromosome segregation [7], and the eukaryotic cilium [8]. Though trypanosomatids have been the focus of many studies, one large and diverse subgroup of plant-infecting trypanosomatids known as *Phytomonas* are relatively poorly understood. Little is known of their biology, life cycle, or how they have adapted to life inside plants. This review discusses what is known about *Phytomonas* in terms of disease biology, morphology, and metabolism in the light of emerging genome resources for this globally distributed group.

## *Phytomonas*: Endophytes and Pathogens

Over 100 years have passed since the first plant trypanosomatids were isolated from the latex of *Euphorbia pilulifera* on the island of Mauritius by Lafont in 1909 [9]. The discovery of these trypanosomatids, initially named *Leptomonas davidi*, was confirmed in *E. pilulifera* in Madras (4,400 kilometres away) later the same year by Donovan [10]. It was recognised from the outset that the plant trypanosomatids were morphologically distinct from mammalian infective species [10], and it was on this basis that Donovan suggested a new genus termed *Phytomonas* for their classification [10]. The first reports of *Phytomonas* infections of plants initially described the host plants as exhibiting poor growth and wilt [9]. Subsequent observations in *Euphorbia segetalis* and *Euphorbia peplus* agreed that infection with *Phytomonas* was deleterious to the plant [11], and reported that infected plants exhibited a depletion of starch granules from the latex and surrounding parenchyma and a reduction in the viscosity of the latex [12]. However, in contrast to this, other authors stated that infections in different Euphorbiaceae produced no detectable effects on plant growth or yield. Moreover, they observed that parasitised and nonparasitised plants were indistinguishable without examination of the latex by microscopy [12,13]. Thus, from the outset there was contention as to whether these parasites were pathogenic or endophytic (nondetrimental) in their plant hosts.

Since these initial investigations into the pathogenicity of *Phytomonas* parasites, two species have been definitively shown to cause plant disease. The first species, *Phytomonas staheli*, is the aetiologic agent of “*hartrot* (fatal wilt) of coconut palm (*Cocos nucifera*), “*marchitez sorpresiva*” (sudden wilt), and slow wilt of oil palm (*Elaeis guineensis*) [14,15], both diseases are acute lethal wilts that begin in the leaves and progress to the rotting of the spear and root [16], although this rotting may be the result of secondary infection by bacteria and other organisms [14]. The second pathogenic species, *Phytomonas leptovasorum*, causes coffee phloem necrosis in both Liberica and Arabica coffee [17]. A common feature of both of these pathogenic *Phytomonas* species is that they exclusively inhabit the phloem during the plant stage of their life cycle [14,17]. A third species, *Phytomonas françai*, inhabits the latex ducts of cassava (*Manihot esculenta*) and has been linked with yield loss diseases known as “*chochamento de raizes*,” or empty root syndrome. This disease is characterised by poor root development and chlorosis of the leaves [18,19]. Several attempts have been made to show experimentally that *P. françai* is the aetiological agent of empty root syndrome; however, in experimental infections, infected plants appeared identical to uninfected individuals [13,19,20]. Moreover, the Unha cultivar of cassava that may have exhibited pathology when infected with *Phytomonas* is no longer widely farmed in Brazil and therefore there have been no reports of empty root syndrome in cassava since 1980 [16]. Thus, it appears that *P. françai* poses little or no risk to food security in this crop.

Though there are only two proven pathogenic species, those species pose some economic risk in South America. *P. leptovasorum* (the aetiological agent of coffee phloem necrosis) has been isolated in Suriname [21] and Brazil [22]. With the rapid expansion of coffee plantations across South America and the resulting change in the distribution of crop pathogens and pests [23], coffee phloem necrosis poses a potential risk to these expanding coffee economies. For example, Brazil is the world’s largest exporter of green coffee beans trading more than 1,700,000 tonnes in 2011 at a value of 8,000 million dollars (http://faostat.fao.org/). *P. staheli* (the aetiological agent of wilt diseases of oil and coconut palm) has previously been isolated from Ecuador, Suriname, Venezuela, Brazil, Costa Rica, Honduras, and Colombia [24]. In these regions, oil palm is a major commercial crop with 397,000 tonnes of palm oil exported by Colombia and Ecuador alone in 2011 at a value of 493 million dollars (http://faostat.fao.org/). Currently, there are no effective treatments of these *Phytomonas* plant diseases, and strategies for infection control comprise the felling of diseased plants or the removal of the infected plant material. Attempts to develop effective chemical controls have suffered from a lack of genome resources and have focused on exploiting targets that are conserved in related trypanosomatid species [25]. These include perturbation of shared metabolic pathways such as aspects of nucleic acid biosynthesis [25].

## Host Range and Phylogeny

**Figure 1:**
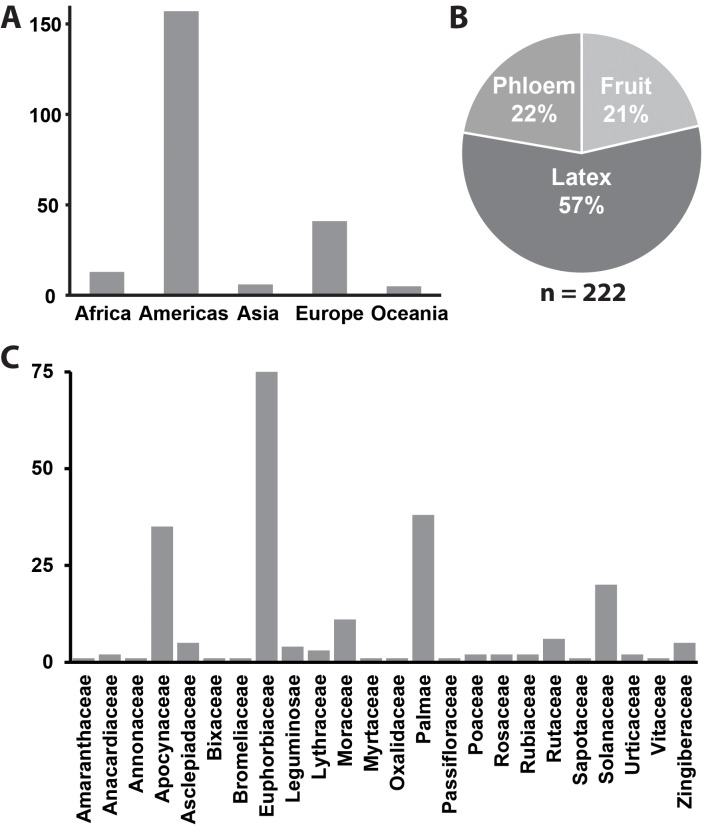
Summary of all known *Phytomonas* isolates. **A**) A bar chart depicting the number of reported isolations by continent. **B)** A pie chart depicting the plant host environment from which these isolations were made. **C)** A bar chart depicting the plant families from which these isolations were made. For further details see S1 Table.

*Phytomonas* as a group are globally distributed (Fig. 1A) and have been repeatedly isolated from disparate plant hosts that span the angiosperm tree-of-life (Fig. 1B). S1 Table is a list of all published *Phytomonas* isolations known at the time of publication of this article and is summarised in Fig. 1. Within their plant hosts *Phytomonas* species have been isolated form a variety of different tissues including phloem, latex ducts, fruit, flowers, and seeds (Fig. 1C & S1 Table) and thus have evolved to inhabit both extracellular and intracellular plant environments [16].

The first attempt to rationalise the phylogeny and subgroupings of *Phytomonas* used isoenzyme analysis [26]. This was extended using molecular markers, including the internal transcribed spacer of the ribosomal RNA locus, kinetoplast DNA sequence, and the spliced leader RNA gene array [27–30]. Collectively, these studies revealed that *Phytomonas* are monophyletic and that the pathogenic phloem-limited *Phytomonas* species formed a discrete group termed the phloemicola that are distinct from nonpathogenic trypanosomatid parasites of fruit and latex [28]. Outside of this association, molecular data revealed that neither habitat nor host species was predictive of phylogenetic subgroupings within *Phytomonas* [30]. Moreover, it was found that the same plant species could harbour distantly related *Phytomonas* species. For example, the genome strain *Phytomonas serpens* 9T isolated from tomato fruit (*Solanum esculentum*) in Brazil belongs to a different group than *Phytomonas* isolated from tomatoes from Southern Spain [30]. Similarly, the *Phytomonas* species isolated from plant latex in Europe, Africa, and India were serologically and molecularly distinct from parasites isolated from latex from South America [30]. However, the low quantities of data available precluded the complete resolution of the evolutionary relationship between all phylogenetic groups.

**Figure 2:**
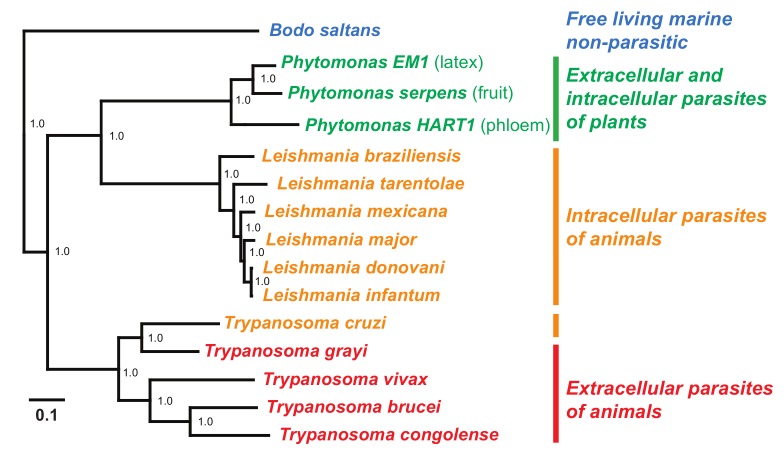
A maximum likelihood protein sequence phylogenetic tree of trypanosomatids. The protein sequence files for a subset of available trypanosomatid genomes were downloaded from TriTrypDB [95]. These were combined with the newly predicted protein sequences from the *Phytomonas* genomes and subjected to clustering using OrthoMCL [96]. Clusters that contained only single copy genes in each of the species were selected (n = 959). These single copy gene families were aligned using MergeAlign [97] and concatenated to form a super alignment. This concatenated alignment was subject to phylogenetic inference using maximum likelihood implemented in the FastTree algorithm [98]. The tree was inferred utilizing the JTT (Jones, Taylor & Thornton) model of amino acid substitution and the CAT model of rate heterogeneity. The topology received 100% support by approximate SH-like support values. Scale bar indicates number of changes per site.

The first *Phytomonas* genomes to be published were those of *P. serpens* 9T [31], *Phytomonas* sp. HART1 [32], and *Phytomonas* EM1 [32]. *P. serpens* 9T was isolated from a tomato fruit in South America [33], *Phytomonas* HART1 was isolated from the phloem of a diseased coconut palm in Guiana [30,32], and *Phytomonas* EM1 was isolated from the latex of an asymptomatic *Euphorbia* in the south of France [30,32]. Fig. 2 is a phylogenetic tree constructed from 959 single copy nuclear genes, adapted from [34] with the inclusion of *L. tarentole, L. donovani, Phytomonas* HART1, and *Phytomonas* EM1, showing the evolutionary relationship of the sequenced *Phytomonas* species to the several pathogenic and nonpathogenic taxa from *Leishmania* and *Trypanosoma*. Interestingly, these *Phytomonas* genomes are much smaller than those of their *Leishmania* or *Trypanosoma* relatives; for example, the genome of EM1 is 17.8 mega bases (Mb) [32] in comparison to 32.9 Mb of *Leishmania major* [35], 62.3 Mb of *Trypanosoma brucei* [36] and 32.5 Mb of *Trypanosoma cruzi* [37]. A unifying feature of phylogenetic analyses of *Phytomonas* is that there appears to be large diversity within the group. For example, the kDNA sequence data shows that the divergence between the phloem limited *Phytomonas* HART1 and the fruit parasite *P. serpens* is greater than that spanning all sampled *Leishmania* species and similar to the divergence between *T. cruzi* and *T. brucei* [32]. This finding is also supported by studies of other molecular markers such as small subunit rRNA and glycosomal GAPDH [38,39] as well as the concatenated protein sequence phylogeny presented in Fig. 2.

## Phytomonas Cell Morphology

Consistent with their sister grouping to *Leishmania*, the majority of *Phytomonas* species isolated to date exhibit promastigote morphology [16,43], characterised by a long cell body with a detached flagellum that protrudes from a flagellar pocket at the cell anterior. This morphology is similar to *Leishmania* parasites in the insect host and many monoxenous trypanosomatid insect parasites. In *Leishmania* and *Trypanosoma*, cell morphologies are replicated with high fidelity through the cell-division cycle and altered with high fidelity when transitioning between different developmental stages depending on the host and organ or type of tissue colonised [43,44]. From the earliest descriptions of *Phytomonas*, it was noted these parasites exhibited extreme morphological polymorphism [11,45]. Polymorphism in cell body width, cell body length, and flagellum length is typical of trypanosomatids isolated from their hosts, common to morphologies with a free (liberform) and attached (juxtaform) flagellum [44]. Whether the observed variation is an adaptation to changes in physiological conditions or uncharacterised life cycle stages is unknown. However, it has been hypothesised [44], based on division of the *Leishmania mexicana* promastigote [46], that one contributing factor to polymorphism of trypanosomatids with a detached flagellum is the pattern of morphogenesis through the cell cycle. In *L. mexicana*, the cell body increases in length during G1 and decreases post-S phase, and flagellar length is highly variable, in particular at cell division where the old flagellum is significantly longer than the new flagellum [46].

Interestingly, *Phytomonas* polymorphism extends beyond promastigote size and shape to include amastigote (no external flagellum) forms. The earliest drawings of parasites isolated from *Euphorbia* latex clearly show cells with a small round cell body and no external flagellum [9,20,47–49] reminiscent of amastigote stages of *Leishmania* parasites [50]. Amastigote-like cells have also been repeatedly extracted from fruit [16]. This is consistent with the hypothesis that there are two major classes of trypanosomatid morphology, those with a free flagellum (including the *Phytomonas* sister taxa *Leishmania*), and those with a laterally attached flagellum, that have conserved pathways for morphogenesis through their cell cycles [44]. However, the biological similarity of these *Phytomonas* amastigote forms to amastigote forms of *Leishmania* parasites and the mechanism(s) regulating differentiation remain completely unknown. In this context it is noteworthy that the functional significance of the promastigote and amastigote cell forms is also unknown, although in *Leishmania* these cell types have been linked with a requirement for motility and sensory functions of the flagellum respectively [51].

## Transmission and Life Cycle

Though trypanosomatids that parasitize fish [52], frogs [53], turtles [53], and possibly several Australian vertebrates [54,55] are transmitted by leeches, the overwhelming majority of described trypanosomatid species are spread between hosts by insects [54,55]. Very shortly after the initial discovery of *Phytomonas*, it was shown in caged insect experiments that the phytophagous insect *Nysius euphorbiae*, a hemipteran that feeds on multiple species of Euphorbia, was able to transmit the parasites from infected plants to uninfected hosts [50]. Further work by Bouet and Rouband (1911) reviewed in [12] demonstrated that the latex-feeding insect *Dieuches humilis* could also act as the insect host; however, the natural host was shown to be the nocturnal coreid spurgebug *Dicranocephalus agilis* [11]. Taken together, these early investigations suggested that individual *Phytomonas* species may be spread between plant hosts by a broad range of different insects. Consistent with these early reports, all subsequent evidence suggests that *Phytomonas* are transmitted by phytophagous insects [45,56,57]. Though the relationship between plant host, parasite, and insect host appears simple (S1 Table), in reality it is unknown to what extent different *Phytomonas* species can be spread by different insects or can colonise different plants. This relationship between plant host, parasite, and insect host is further complicated by the nomenclature of the parasites. For example, the name *P. serpens* has been applied to *Phytomonas* parasites that have been isolated from the fruit of multiple different tomato cultivars (S1 Table) in the Americas, Africa, and Europe [30,45]. Moreover, it has also been applied to isolates from multiple different insects, for example, from the salivary glands of *Nezara viridula* in South Africa [45] and from *Phthia picta* in Brazil [58]. These multiple disparate isolations may represent different species and thus there may be some inflation of the true host and insect host range for an individual species.

In insect-spread mammalian infections of *Leishmania* and *Trypanosoma*, parasite cells undergo differentiation to metacyclic forms that are capable of infecting the mammalian host. In *Trypanosoma brucei*, these infective metacyclic cells express a specialised variant surface glycoprotein on their cell surface [59] while in *Leishmania major*, the molecular marker for mammalian infective metacyclic cells is a specialised lipophosphoglycan (LPG) [60,61]. There is no evidence for metacyclic variant surface glycoproteins or metacyclic LPG in *Phytomonas*. Moreover, the metacyclic specific LPG found in *L. major* is not found in other *Leishmania* species, and there are no other known molecular markers of metacylogenesis. Though there are descriptions of metacyclic *Phytomonas* cells in the literature [45], these descriptions are based solely on cell morphology and it is unknown whether they represent a true specialised plant infective stage of the life cycle. In this context it is noteworthy that there have been successful infections of tomato fruit with insect isolated *P. serpens* cells grown in culture and from allowing infected insects to feed on fruit [45,62]. This is in contrast to *Leishmania* and *Trypanosoma* where cultured insect adapted cells are not infective to mammalian hosts and metacylogenesis must be induced prior to infection [63]. Though a preadapted metacyclic stage may not be present in the *Phytomonas* life cycle, *P. serpens* cells have been observed to attach to insect salivary glands [56] indicative of a true developmental stage inside the insect host.

Some studies have begun to emerge on the effect of *Phytomonas* infection on insect hosts. It has been demonstrated that *P. serpens* infection is countered by phagocytosis by hemocytes in the milkweed bug *Oncopeltus fasciatus* [56]. However, the responses of the hemocytes were not sufficient to prevent the parasite from attaching to the salivary glands within 24 hours post infection [56]. Future studies may reveal whether infection of insect hosts leads to alteration in host behaviour. For example, *Leishmania* infection of the sandfly *Lutzomyia longipalpis* results in increased biting persistence and a concomitant increase in transmission efficiency [64]. Similarly, some bacterial pathogens increase their transmission efficiency by modification of their plant host. For example, infection with some *Phytoplasma* species can cause yellowing of infected plant tissue resulting in increased transmission as phloem-feeding insects prefer young green/yellow leaves [65]. It has yet to be shown whether *Phytomonas* infection leads to alteration of plant or insect host for enhanced transmission. However, emerging data suggests that *Phytomonas serpens* is able to produce auxin and thus may interfere with plant hormone signalling [66].

## The *Phytomonas* Cell Surface

**Figure 3:**
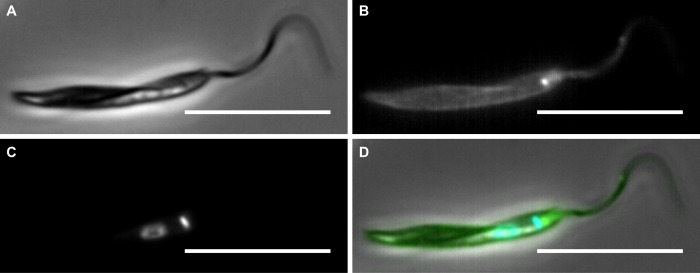
Epifluorescence microscopy to show cell surface biotin labelling of *Phytomonas serpens*. Cells were labelled with EZ-link-sulfo-NHS-SS-Biotin (Thermo Scientific) and fixed in 4% paraformaldehyde. Biotin was detected using streptavidin AlexaFlour488 (green) (Invitrogen). DNA was stained using Hoechst (blue). **A)** Phase contrast image of a *P. serpens* cell. **B)** Distribution of biotin on the cell surfaces to show at least three cell membrane domains. **C)** DNA. **D)** Merge of all channels. Scale bar 10μm.

The cell surface of *Phytomonas*, like all trypanosomatids, consists of at least three discrete domains. The domain with the largest surface area is the cell body surface; there is also the flagellar surface and next to the flagellum a distinct portion of the cell body membrane that is invaginated to form the flagellar pocket. In all trypanosomatids, these cellular surfaces lack an outer cell wall and instead are coated with membrane-anchored proteins and glycoinositol phospholipids [67]. This exterior cell surface acts to protect the parasite cell from host responses and from changing environmental conditions. The three domains are visible when whole *Phytomonas* cells are labelled with biotin and incubated in the presence of a streptavidin conjugated fluorescent dye as shown in Fig. 3.

To date, few molecular components of the *Phytomonas* cell surface have been characterised. Those that have been so far identified are those that are conserved in other trypanosomatids. One major component is the zinc-metalloprotease Major Surface Protease (MSP), also known as gp63 [68]. This protein is likely to be pan-eukaryotic [69] and is found ubiquitously among the trypanosomatids in both a secreted form and a form anchored to the plasma membrane by glycosylphosphatidylinsoitol (GPI) [70,71]. The role of gp63 in the plant host has yet to be interrogated; however, there is some evidence to suggest that gp63 is important in the insect stage of the *Phytomonas* life cycle [68]. Treatment of *P. serpens* with antibodies raised to gp63 perturbs parasite morphology and results in loss of adhesion to the salivary glands of the insect host, a step necessary for the establishment of infection in the insect host [33,72]. Along with gp63, cysteine-proteases related to cruzipain of *T. cru*zi have also been found in *P. serpens* [33]. Both of these proteins have been shown to be secreted by *P. serpens*, as well as existing in a form anchored to the cell surface by GPI [72]. Consistent with this observation, interrogation of the *Phytomonas* genomes revealed the presence of the pathways for GPI anchor biosynthesis [32].

Analysis of the genome sequences also revealed the surprising finding that HART1 contains an expansion in the gp63 gene family with over 20 members in contrast to 2 genes in EM1 [32]. This expansion suggests that HART1 may have a more complex cell surface that might facilitate survival in the hostile environment of the plant phloem [73,74]. This increased diversity may also be further enhanced through post-translational modification as the genes required for post-translational addition of glucose, mannose, galactose, N-acetylglucosamine, glucuronic acid, xylose, and fucose are also present in one or both *Phytomonas* genomes [32]. While the function of these surface proteins in either the plant or insect host have yet to be established, it has been shown that the expression of these proteins in *Phytomonas* is responsive to culture conditions [75]. For example, incubation in media-containing high levels of protein causes expression of GP63 to decrease [75]. Expression also correlates with growth rate, and therefore it may play a role in allowing the *Phytomonas* parasites to adapt to different environments [72].

In addition to proteins, trypanosomatids express a rich and varied array of glycolipids on their cell surface. *Leishmania* cells, for example, are coated in a layer of lipophosphoglycan (LPG) that helps to protect the parasite from host pressures such as complement-mediated lysis in mammals [76]. While LPG has yet to be isolated from *Phytomonas*, the complete enzyme complement for the synthesis of LPG is present in the *Phytomonas* genomes [32] and thus LPG is likely to be present at the cell surface. Moreover, these genes may also function in the biosynthesis of other classes of structurally related glycolipids known as glycoinositol phospholipids (GIPLs). Interestingly, it has been shown that the major glycolipids of the *Phytomonas* cell surface are GIPLs [67]. Four different GIPLs have been characterised from a *Phytomonas sp*. isolated from *Euphorbia characias* and collectively these GIPLs are present at ≥ 5 ×10^6^ copies per cell [67]. The function of these highly abundant surface molecules is as yet unknown; however, one hypothesis is that the dense negative charge they give to the cell surface may provide some protection to the *Phytomonas* parasites in the plant host [67]. Specifically, we propose that in *Phytomonas* the dense negative charge may help repel negatively charged hydroxide ions that are produced by the plant host as part of the oxidative burst; a rapid, transient production of reactive oxygen species that is one of the first responses in a plant’s defence strategy [77]. It may be that the GIPLs function together with other cell surface glycolipids and glycoproteins in this role, for example in *Leishmania* there is evidence to show that LPG provides some protection against reactive oxygen species [78,79]. The specific composition of GIPLs in *Phytomonas* may also be important for adaptation to different plant hosts. For instance, in one *Phytomonas* strain, it was found that the GIPLs lack galactose residues that are common in Leishmania GIPLs [67]. As plants express a diverse array of lectins with affinities for different hexose moieties, it may be that lectins from some plant species will effectively agglutinate individual *Phytomonas* species while others will not [67]. For example, the latex of the host plant (*Euphorbia characias*) from which the *Phytomonas* strain above was isolated, contains a galactose-specific lectin [80] that may agglutinate any parasite expressing galactose on its cell surface [67]. Though this hypothesis was not tested in the plant host, a galactose-specific lectin from a different lactiferous plant (*Ricinus communis* RCA1) resulted in effective agglutination of the same species of *Phytomonas* [81]. Further investigation will reveal whether the composition of GPILs play a role in plant host specificity.

Intriguingly, immunogenic similarities between *P. serpens* and *T. cruzi* are enough that oral exposure to *P. serpens* has been observed to attenuate the symptoms of Chagas disease in C57BL/6 mice [82]. It is also interesting to note that while naturally occurring *Phytomonas* infections in mammals have not been described, inoculation of mice with infected latex has been reported to produce sustained infections [83]. Following inoculation, it was noted that the spleen was increased in volume and that blood smears from this organ contained amastigote-like forms of the parasites. It may be that some species of *Phytomonas* are capable of infecting mammals but that their host range is governed by the feeding habits of their invertebrate host. However, caution should be exercised here as the parasites used were identified solely on the basis of their presence in plant latex, and this may have occurred through infection from other opportunistic trypanosomatids. Furthermore, there have yet to be reports of animal infections with *Phytomonas* in wild populations. Taken together, this suggests that if animal infections do exist, either they do not persist or retransmission does not occur.

One particularly poorly understood aspect of *Phytomonas* biology is whether they secrete protein effectors to modulate the host plant immune response, an adaptation commonly found in other plant parasites [40]. A genome-wide analysis of the predicted *Phytomonas* EM1 and HART1 secretomes was undertaken in the hope that it would lead to the identification of potential pathogenicity effectors in *Phytomonas* parasites. This resulted in the identification of several aspartyl proteases in both *Phytomonas* strains that were absent from the genomes of *Leishmania* and *Trypanosoma* [32]. These proteases may play a role in host–parasite interactions as phloem sap often contains aspartyl protease inhibitors, and aspartyl proteases are known to be secreted by plant pathogenic fungi such as *Botrytis cinerea* [41] and have been implicated in the suppression of systemic acquired resistance [40]. However, the role of these inhibitors has so far mainly been investigated in the context of phloem-feeding herbivores [42], and there is some doubt whether these proteases are involved in plant–fungal interactions as protease deficient strains of *B. cinerea* are not attenuated in virulence [41].

Comparative surface proteomics between *Phytomonas, Leishmania*, and *Trypanosoma* may yield further insight into the molecular reasons for these immunogenic similarities. Moreover, it may help to elucidate the evolution of these disparate parasite cell surfaces and identify factors that distinguish plant-adapted from mammalian-adapted trypanosomatids and facilitate colonisation of different plant environments.

## Phytomonas Metabolism

Analysis of the genes encoding metabolic proteins in the *Phytomonas* genome sequences revealed a cohort of enzymes consistent with life in a plant environment. Both HART1 and EM1 genomes encode glucoamylase, alpha-glucosidase, and alpha, alpha-trehalose phosphorylase (acquired through horizontal gene transfer) genes, allowing them to utilise plant carbohydrates [32]. Interestingly, only the phloem restricted pathogen, *Phytomonas* HART1, encodes invertase genes for degradation of sucrose [32]. This finding is consistent with the diverse metabolic profiles of fruit [84] and latex [85] compared to the overwhelming abundance of sucrose in the phloem. In addition, the *Phytomonas* genomes contain few tandemly duplicated genes in comparison to *Trypanosoma* and *Leishmania* species where the genes encoding metabolic enzymes are frequently found as tandem duplicates [32].

Another remarkable feature of *Phytomonas* parasites is the loss of genes encoding the cytochrome *c* oxidase subunits I–III (COI, COII, COIII), and cytochrome b (CyB) of the bc1 complex [32,86,87]. This loss appears to have occurred at the base of the *Phytomonas* clade [31]. One aspect of *Phytomonas* biology that may have facilitated the loss of the cytochromes is the feeding behaviour of the insect host. Phytophagous insects feed exclusively on carbohydrate rich plant sap and therefore there may be no requirement for a metabolic shift from a carbohydrate to an amino acid energy substrate [32]. In the case of the former, adenosine triphosphate (ATP) production can occur at the level of substrate phosphorylation [88], but in the case of the latter, the mitochondrial respiratory chain is required for the complete oxidation of the substrate [36]. The absence of a requirement to use amino acids as the main source of energy for *Phytomonas* in the insect host may have contributed to the loss of the mitochondrial cytochromes and the sole production of ATP via glycolysis in glycosomes [32,86,87,89,90]. In this regard, it will be interesting to see whether these metabolic differences manifest changes in the enzyme content of glycosomes in *Phytomonas* in comparison to other trypanosomatids.

An essential cofactor for the mitochondrial cytochromes is heme, an iron molecule contained by a porphyrin ring, which functions in redox reactions and electron transport [91]. Interrogation of the available *Phytomonas* genomes and the culture of *P. serpens* in a heme-free medium suggests that they are the only aerobic eukaryotes with the ability to survive in the absence of this molecule [31]. However, the relationship between these plant trypanosomatids and heme is not straightforward, as *P. serpens* cells grown in the presence of heme did contain small amounts of the compound, indicating that they are able to scavenge heme from the medium [31]. To further complicate this curious aspect of *Phytomonas* biology, the coding sequence for the enzyme ferrocheltase (involved in heme synthesis) is present in *P. serpens*, and sequences encoding two heme-containing proteins are found in all three available *Phytomonas* genomes [31].

In addition to the alpha, alpha-trehalose phosphorylase mentioned above, there is also at least one other instance of horizontal gene transfer that conferred novel metabolic capabilities to *Phytomonas*. The transferred gene encodes a zinc-dependant alcohol dehydrogenase [92,93], which in combination with malate dehydrogenase enables *Phytomonas* species to produce lactate [32,94]. It is unknown whether the production of lactate may produce a selective advantage for *Phytomonas* within the plant or insect host.

## Discussion and Future Perspectives

*Phytomonas* are an apparently ubiquitous and diverse group of plant parasites exhibiting both pathogenic and endophytic lifestyles. From their initial discovery in 1909 to the publication of the first *Phytomonas* genomes over 100 years later, much progress has been made in their classification, taxonomy, and aspects of cell biology.

The apparent abundance of endophytes in the genus *Phytomonas* may be representative of the extant diversity, or may be the result of a sampling bias. The first plant trypanosomatids were first discovered in the latex of a Mediterranean spurge, and as a result the bulk of subsequent sampling was conducted in the plant family *Euphorbiaceae* [9,16]. The apparent lack of disease causing *Phytomonas* may simply be due to the fact that *Euphorbia*-infecting *Phytomonas* are generally asymptomatic. Thus, increased sampling needs to be completed before broad conclusions about the diversity and pathogenicity in this genus are made. In this context, it is important to bear in mind that the breadth of the host range (both plant and insect) for any individual *Phytomonas* species is unknown, and many aspects of the life cycle are poorly understood. While different cell types and life cycle stages have been described in the literature [45], molecular markers for these stages have yet to be identified, and it is unknown whether cells differentiate to adapt to plant and insect hosts and to what extent both host stages are necessary to complete the life cycle.

The wide morphological diversity exhibited by this genus of plant-infecting flagellates is still poorly understood in an adaptive context. In other trypanosomatids, changes in cell morphology represent different life cycle stages, and cell shape, and form is the product of the different selection pressures associated with the discrete host environments colonised [44]. Further studies concerning *Phytomonas* cell morphology during the cell cycle could address the question as to whether the diverse morphology seen in infections is simply a product of the cell division cycle or is adaptive and bears implications for virulence.

The release of the first *Phytomonas* genomes has already begun to reveal clues into their biology and pathogenicity. For example, the expansion of a gene family of putative secretable aspartyl proteases in the pathogenic isolate is reminiscent of similar phenomena in plant pathogenic fungi [32]. It will be interesting to see whether the publication of these novel genome resources will help elucidate mechanisms of pathogenicity in *Phytomonas* and resolve whether disease symptoms in host plants arise as a result of a parasite mediated degradation of plant tissues or as an indirect by-product of depletion of plant assimilates. In either case, it is likely that surface proteins play a role in the adaptation of *Phytomonas* to their plant hosts because of the involvement of such proteins in environmental sensing and parasite–host interactions. Moreover, there is abundant precedent in *Leishmania* and *Trypanosoma* that parasite surface proteins play a crucial role in mediating parasite–host interactions and in evading host immune responses. Thus, it is likely that characterisation of a *Phytomonas* surface-localised and-secreted proteome may yield insights into both disease biology and the evolution of parasite surfaces in the trypanosomatids. This may also provide an avenue for the identification of the factors necessary for the invasion of plant tissue and the development of systemic infection.

Taken together, the emergence of genome resources will provide a new foundation for discovery in this enigmatic and globally-distributed group of plant parasites. Moreover, it will provide new insight into the evolution of the trypanosomatids and how these intriguing parasites now inhabit a host range that spans from crocodiles to coconuts.

## Supporting Information

S1 Table List of all previously reported isolations of *Phytomonas* parasites, including plant and insect host species, plant family, country of isolation and citation where possible. (XLSX)
