# Correction: *Phytomonas*: Trypanosomatids Adapted to Plant Environments

**DOI:** 10.1371/journal.ppat.1004927

**Published:** 2015-05-19

**Authors:** Eleanor Jaskowska, Claire Butler, Gail Preston, Steven Kelly

Fig 1 does not include axis labels and has an outdated plant family name (Palmae). The authors have provided a corrected version here.

**Fig 1:**
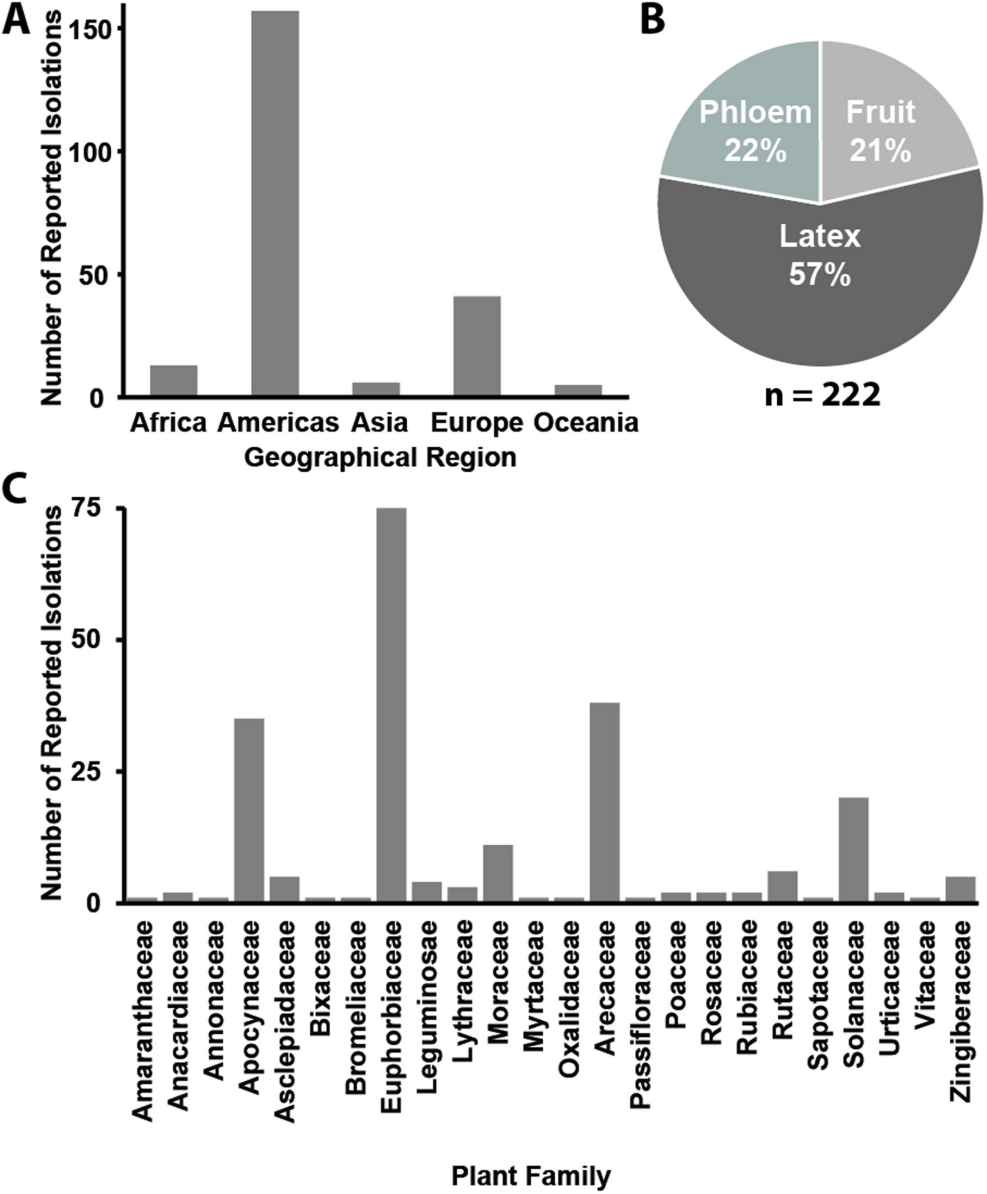
Summary of all known *Phytomonas* isolates. A) A bar chart depicting the number of reported isolations by continent. B) A pie chart depicting the plant host environment from which these isolations were made. C) A bar chart depicting the plant families from which these isolations were made. For further details see S1 Table.
